# Mesenteric Lymph Drainage Alleviates Acute Kidney Injury Induced by Hemorrhagic Shock without Resuscitation

**DOI:** 10.1155/2014/720836

**Published:** 2014-02-25

**Authors:** Zi-Gang Zhao, Hong-Xia Zhu, Li-Min Zhang, Yu-Ping Zhang, Chun-Yu Niu

**Affiliations:** Institute of Microcirculation, Hebei North University, Diamond South Road 11, Zhangjiakou, Hebei 075000, China

## Abstract

This study aimed to investigate the effect of mesenteric lymph drainage on the acute kidney injury induced by hemorrhagic shock without resuscitation. Eighteen male Wistar rats were randomly divided into sham, shock, and drainage groups. The hemorrhagic shock model (40 mmHg, 3 h) was established in shock and drainage groups; mesenteric lymph drainage was performed from 1 h to 3 h of hypotension in the drainage group. The results showed that renal tissue damage occurred; the levels of urea, creatinine, and trypsin in the plasma as well as intercellular adhesion molecule-1 (ICAM-1), receptor of advanced glycation end-products (RAGE), tumor necrosis factor-**α** (TNF-**α**), malondialdehyde (MDA), lactic acid (LA), and 2,3-DPG in the renal tissue were increased in the shock group after 3 h of hypotension. Mesenteric lymph drainage lessened the following: renal tissue damage; urea and trypsin concentrations in the plasma; ICAM-1, RAGE, TNF-**α**, MDA, and LA levels in the renal tissue. By contrast, mesenteric lymph drainage increased the 2,3-DPG level in the renal tissue. These findings indicated that mesenteric lymph drainage could relieve kidney injury caused by sustained hypotension, and its mechanisms involve the decrease in trypsin activity, suppression of inflammation, alleviation of free radical injury, and improvement of energy metabolism.

## 1. Introduction

In the pathogenesis of hemorrhagic shock induced by trauma, operative accidents, traffic accidents, and earthquakes, the kidney is one of the organs, in which hypoperfusion initially occurs after hemorrhage because the sympathetic-adrenal medulla system and the renin-angiotensin-aldosterone system are activated. With the development of fluid resuscitation based on microcirculation disturbance theory, the cases of kidney injury induced directly by ischemia following shock decline gradually [1]. Under specific circumstances, a large proportion of shocked patients receive delayed fluid resuscitation because factors inducing hemorrhage are complex [2, 3]. Therefore, acute kidney injury (AKI) following hemorrhagic shock remains a serious problem. AKI occurs in approximately 30% of patients admitted in intensive care units and is commonly associated with multiple-organ dysfunction syndrome (MODS) [4]. Hence, related mechanisms should be elucidated to develop intervention treatment for kidney injury caused by sustained hypotension.

Previous studies found that the intestinal lymphatic pathway has an important function in the pathogenesis of MODS; posthemorrhagic shock mesenteric lymph return is a major factor causing multiple-organ injury [5–7]. Our studies showed that the mesenteric lymph duct ligation (MLDL) could alleviate kidney injury following two-hit of hemorrhage and lipopolysaccharide and hemorrhagic shock with fluid resuscitation [8, 9]. However, further studies should be conducted to determine whether or not the blockage of mesenteric lymph return can decrease kidney injury after hemorrhagic shock without resuscitation. A few limitations have been documented in the gap between MLDL and clinical application. Therefore, the current study investigated the effect of mesenteric lymph drainage on AKI induced by hemorrhagic shock without resuscitation. This study also determined the related mechanisms in terms of trypsin activity, inflammatory response, energy metabolism, and other factors. Furthermore, this study aimed to (1) clarify the significance of posthemorrhagic shock mesenteric lymph in the pathogenesis of AKI and (2) provide a convictive theoretical and experimental evidence for the clinical interference of severe shock.

## 2. Methods

### 2.1. Animals

Eighteen adult male-specific pathogen-free Wistar rats weighing 250 g to 300 g (Chinese Academy of Medical Sciences Animal Breeding Center) were used in the experiments. The rats were randomly divided into the following (*n* = 6 in each group): sham group; shock group (hemorrhagic shock model); and drainage group (hemorrhagic shock plus mesenteric lymph drainage). The rats were maintained in accordance with the National Institutes of Health Guide for the Care and Use of Laboratory Animals, and the research protocol was approved by the Institutional Animal Use and Care Committee of Hebei North University.

### 2.2. Experimental Model

The rats were anesthetized with pentobarbital sodium (50 mg/kg). Under aseptic conditions, all of the rats received femoral operations to isolate the right femoral vein and bilateral femoral arteries for anticoagulation, hemorrhage, and monitoring of mean arterial pressure (MAP) as previously reported [10, 11]. Afterward, the rats were subjected to abdominal dissection to separate the mesenteric lymph duct from the surrounding connective tissues to induce mesenteric lymph drainage. After a stabilization period of 30 min, the hemorrhagic shock model (40 mmHg, 3 h) was established in shock and drainage groups; mesenteric lymph drainage was performed from 1 h to 3 h of hypotension in the drainage group as previously described [10, 12]. At the same time, the rats in the sham group were anesthetized and received femoral and abdominal surgery as well as shock and drainage groups but did not undergo hemorrhage.

### 2.3. Collection of Samples

At 3 h of hypotension or corresponding time, under deeply anesthetic conditions, blood sample of 3 mL was drawn from the abdominal aorta and the plasma was collected by centrifugation at 850 g for 10 min and was stored at −75°C in a refrigerator (Thermo Electron, Waltham, MA) to examine renal function indices and trypsin activity. The renal tissues of the rats were then collected. Subsequently, the left kidney was cut by a longitudinal midline incision; half of the left kidney, including the medulla and the cortex, was fixed in 10% neutral buffered formalin to observe renal morphology. The right kidney was homogenized in 1 : 9 (w/v) physiological saline for 30 s by using the FJ-200 type high-speed tissue homogenizer (Shanghai Specimen and Model Factory, Shanghai, China) and then centrifuged at 850 ×g at 0°C to 4°C for 10 min with the Labofuge 400R supercentrifuge (Heraeus, Hanover, Germany). Supernatant fluids were frozen at −75°C for further assays.

### 2.4. Observation of Renal Morphology

The renal tissue was fixed in formalin, dehydrated in alcohol gradient, and embedded in paraffin. The paraffin-embedded renal tissue was sectioned at 5 *μ*m and stained with hematoxylin and eosin (HE). Morphological changes in the kidney were observed using a light microscope (90i, Nikon, Tokyo, Japan) and photographed using an image collection and analysis system (Eclipse, Nikon, Tokyo, Japan).

### 2.5. Examination of Renal Function

Urea and creatinine (Cre) contents in the plasma were examined by an automatic biochemical analyzer (Aeroset, Abbott, Chicago, IL), and the reagents were purchased from Randox Laboratories Ltd. (Shanghai, China).

### 2.6. Examination of Trypsin Activity

Considering that the ester chains of L-arginine ethyl ester dihydrochloride are hydrolyzed by trypsin, trypsin activity in the plasma was determined using the hydrolysis method according to the manufacturer's instructions (Jiancheng Biotechnology Research Institute, Nanjing, China). One unit of trypsin activity corresponds to the amount of enzyme that produced an increased absorbance of 0.003 per min at 253 nm at pH 8.0 and 37°C.

### 2.7. Examination of Lactic Acid (LA) Concentration

LA concentration in the renal tissue was determined using the dehydrogenation method according to the manufacturer's instructions [13]. The reagent was purchased from Jiancheng Biotechnology Research Institute (Nanjing, China). The result of LA is shown as nanomoles per milligram of protein. The protein of the homogenate was quantified using the Coomassie brilliant blue colorimetric method [13, 14].

### 2.8. Examination of Malondialdehyde (MDA)

MDA concentrations in the renal homogenate were determined by modified thiobarbituric acid microdetermination method according to previous reports [15, 16]. The reagent was purchased from Jiancheng Biotechnology Research Institute (Nanjing, China). The result of MDA is shown as nanomoles per milligram of protein.

### 2.9. Enzyme-Linked Immunoadsorbent Assay (ELISA)

To draw a standard curve, we determined the concentrations of the intercellular adhesion molecule-1 (ICAM-1), advanced glycation end-product receptor (RAGE), tumor necrosis factor-*α* (TNF-*α*), and 2,3-DPG by ELISA according to the manufacturer's instructions (antibodies were purchased from R&D Systems, USA). The concentrations of ICAM-1, RAGE, TNF-*α*, and 2,3-DPG were expressed as per milligram of protein.

### 2.10. Statistical Analysis

Experimental data were expressed as mean ± SD, and statistical analysis was performed using SPSS software 16.0 (Polar Engineering and Consulting Inc., Chicago, IL). One-way ANOVA was used between groups and Student-Newman-Keuls (SNK) test was used within groups. *P* < 0.05 was considered significantly different.

## 3. Results

### 3.1. Changes in Renal Pathomorphology

**Figure 1:**
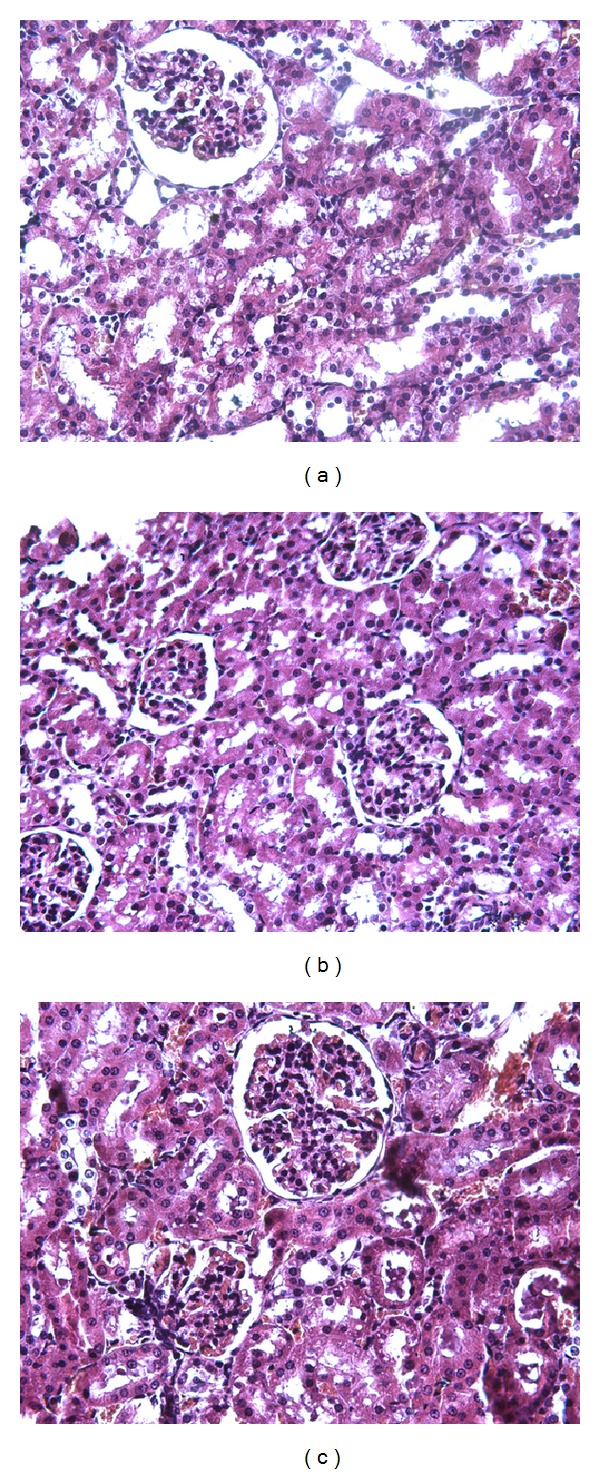
Effects of mesenteric lymph drainage on pathomorphology of kidney in hemorrhagic shock rats without resuscitation (HE staining, ×500). (a) Sham group; (b) shock group; (c) drainage group.

The rats in the sham group exhibit a normal architecture in the renal glomerulus and tubules with clear and distinctive proximal and distal convoluted tubules (Figure 1(a)). Epithelial cell necrosis in the renal tubule was observed in the rats with hemorrhagic shock but without resuscitation (Figure 1(b)). By contrast, mesenteric lymph drainage from 1 h to 3 h of hypotension significantly alleviated the hemorrhage-induced morphological injury (Figure 1(c)).

### 3.2. Change of Renal Function Indices in Plasma

**Figure 2:**
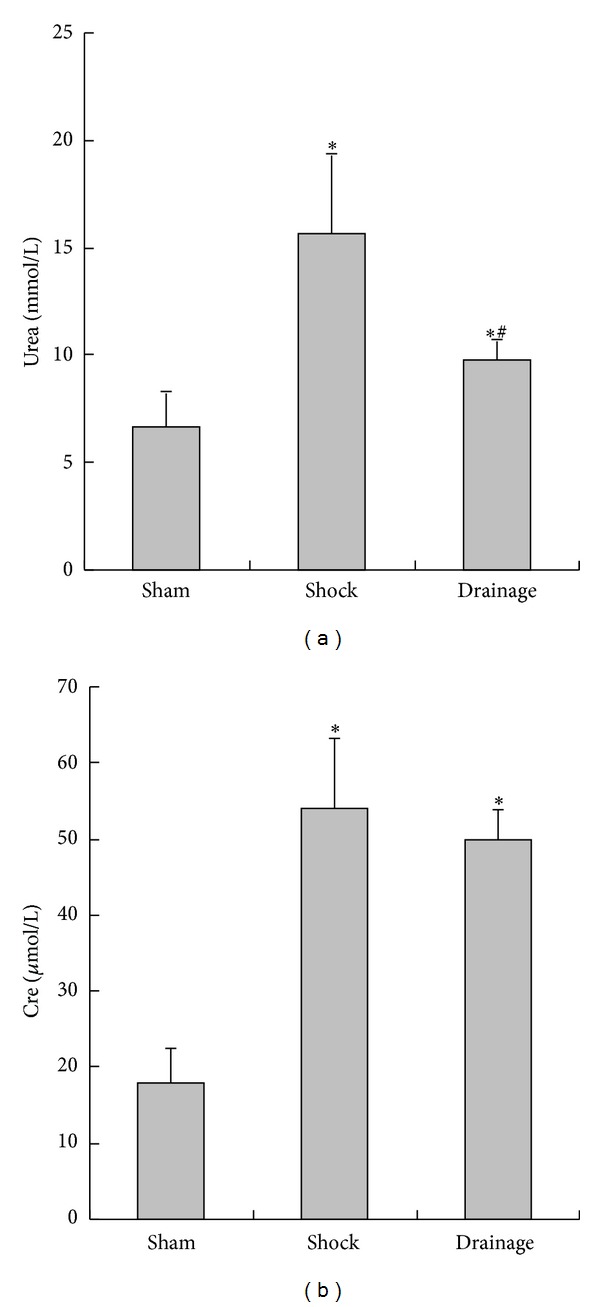
Effects of mesenteric lymph drainage on renal function indices in hemorrhagic shock rats without resuscitation (mean  ±  SD, *n* = 6). *P < 0.05 versus sham group; ^#^ *P* < 0.05 versus shock group.

At 3 h of hypotension, urea and Cre concentrations in the plasma of the rats in shock and drainage groups were significantly higher than those in the sham group; however, the urea concentration in the drainage group was lower than that in the shock group (Figure 2).

### 3.3. Change in Renal Trypsin Activity in the Plasma

**Figure 3:**
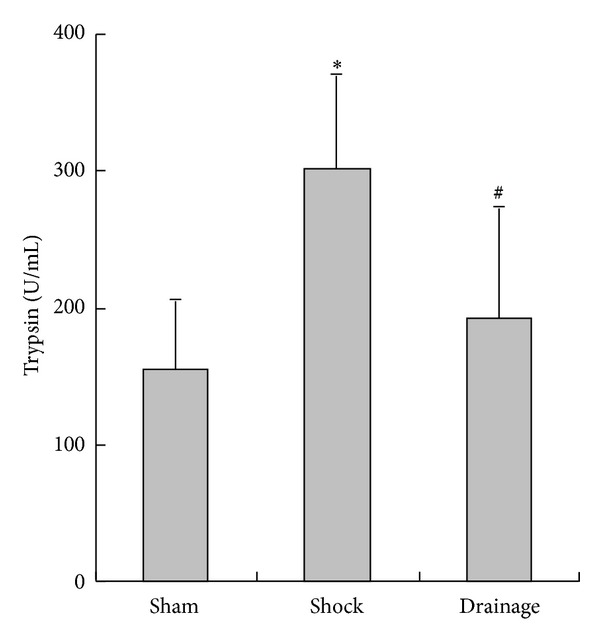
Effects of mesenteric lymph drainage on trypsin activity of plasma in hemorrhagic shock rats without resuscitation (U/mL, mean  ±  SD, *n* = 6). *P < 0.05 versus sham group; ^#^ *P* < 0.05 versus shock group.

Figure 3 shows that trypsin activity in the plasma of the rats in the shock group was significantly increased compared with that in the sham group; the index in the drainage group was decreased compared with that in the shock group, no statistical difference was observed in the sham group.

### 3.4. Change of LA Content in the Renal Homogenate

**Figure 4:**
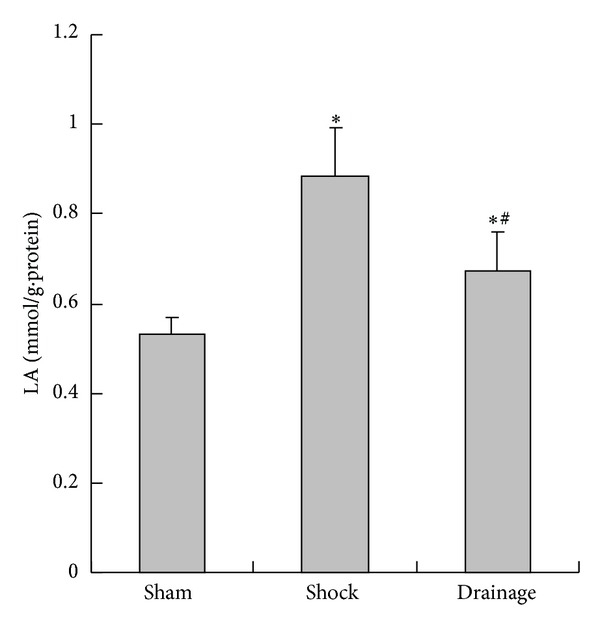
Effects of mesenteric lymph drainage on LA content of renal homogenate in hemorrhagic shock rats without resuscitation (mmol/g·protein, mean  ±  SD, *n* = 6). *P < 0.05 versus sham group; ^#^ *P* < 0.05 versus shock group.

Figure 4 indicates that the LA concentrations in the renal tissue in the shock and drainage groups were significantly increased compared with those in the sham group; nevertheless, LA concentrations in the drainage group were decreased than those in the shock group.

### 3.5. Change of 2,3-DPG Content in the Renal Homogenate

**Figure 5:**
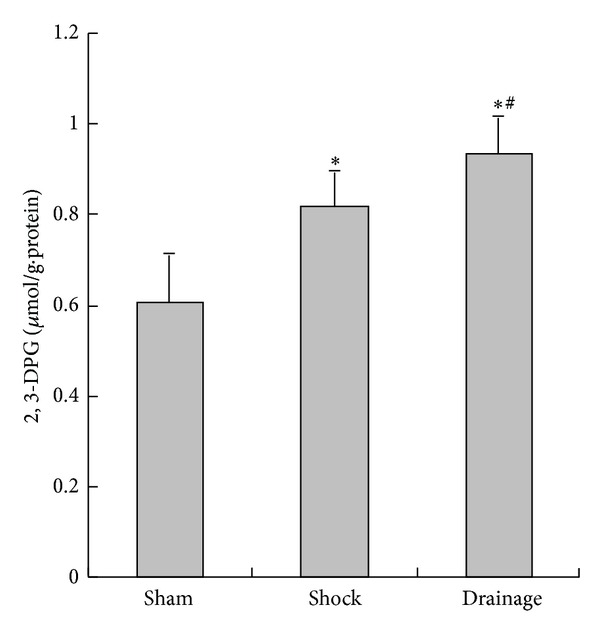
Effects of mesenteric lymph drainage on 2,3-DPG content of renal homogenate in hemorrhagic shock rats without resuscitation (*μ*mol/g·protein, mean  ±  SD, *n* = 6). *P < 0.05 versus sham group; ^#^ *P* < 0.05 versus shock group.

The 2,3-DPG concentration in the renal tissue of the shock and drainage groups were significantly increased than that in the sham group; the index in the drainage group was higher than that in the shock group (Figure 5).

### 3.6. Change of MDA Content in the Renal Homogenate

**Figure 6:**
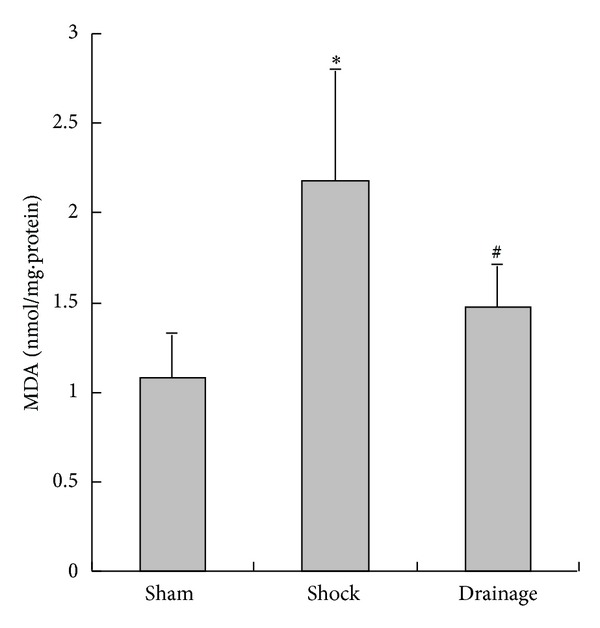
Effects of mesenteric lymph drainage on MDA content of renal homogenate in hemorrhagic shock rats without resuscitation (nmol/mg·protein, mean  ±  SD, *n* = 6). *P < 0.05 versus sham group; ^#^ *P* < 0.05 versus shock group.

The MDA concentrations in the renal tissue of the rats in the shock group were increased compared with those in the rats of the sham group; the MDA concentrations in the renal tissue of the rats in the drainage group were decreased than those in the rats of the shock group. No statistical difference was observed between the drainage and sham groups (Figure 6).

### 3.7. Change in ICAM-1 Concentration in the Renal Homogenate

**Figure 7:**
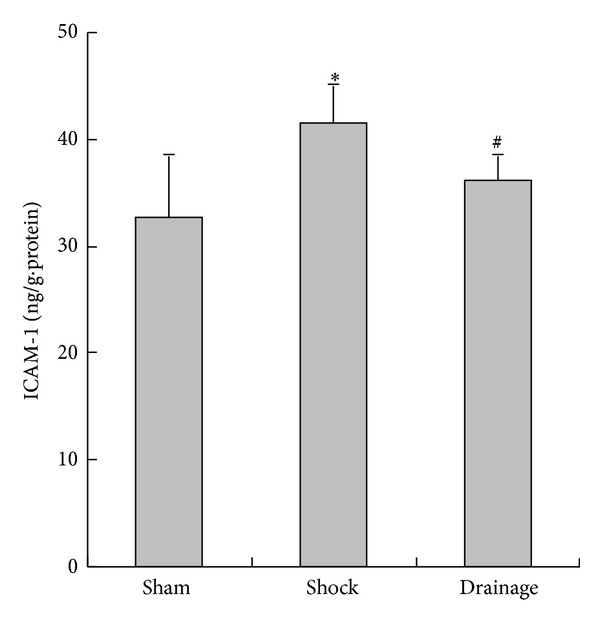
Effects of mesenteric lymph drainage on ICAM-1 contents of renal homogenate in hemorrhagic shock rats without resuscitation (ng/g·protein, mean  ±  SD, *n* = 6). *P < 0.05 versus sham group; ^#^ *P* < 0.05 versus shock group.

Figure 7 shows that the ICAM-1 concentration in the renal homogenate in the shock group was higher than that in the sham group. The ICAM-1 concentration in the drainage group was lower than that in the shock group. No statistical difference was observed with that in the sham group.

### 3.8. Change in RAGE Concentration in the Renal Homogenate

**Figure 8:**
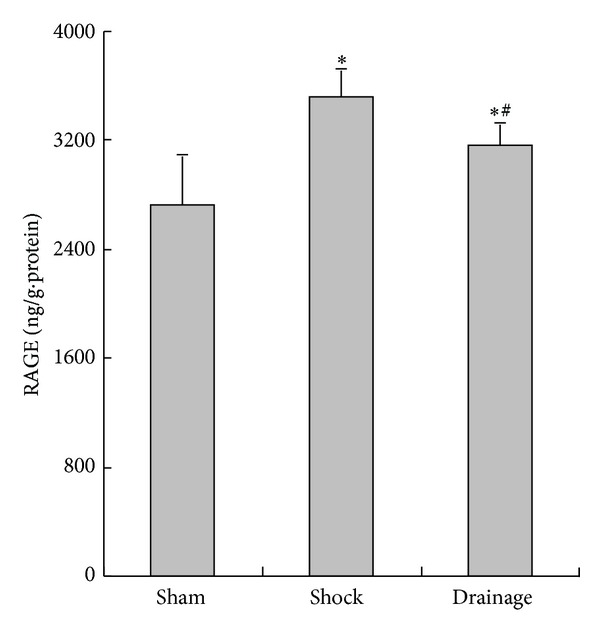
Effects of mesenteric lymph drainage on RAGE contents of renal homogenate in hemorrhagic shock rats without resuscitation (ng/g·protein, mean  ±  SD, *n* = 6). *P < 0.05 versus sham group; ^#^ *P* < 0.05 versus shock group.

The RAGE concentration in the renal tissue of the rats in the shock group was significantly increased compared with that in the sham group. However, the RAGE in the renal tissue of the rats in the drainage group was lower than that in the shock group but higher than that in the sham group, respectively (Figure 8).

### 3.9. Change in TNF-*α* Concentration in the Renal Homogenate

**Figure 9:**
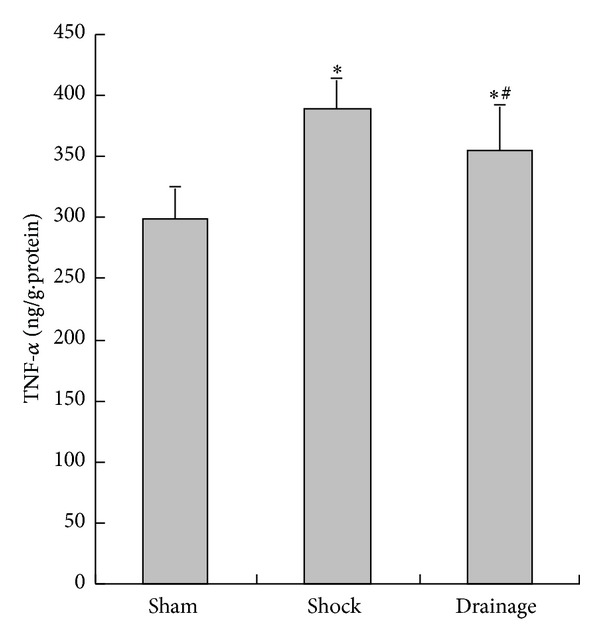
Effects of mesenteric lymph drainage on TNF-*α* content of renal homogenate in hemorrhagic shock rats without resuscitation (ng/g·protein, mean  ±  SD, *n* = 6). *P < 0.05 versus sham group; ^#^ *P* < 0.05 versus shock group.

After the rats were subjected to hemorrhagic shock, the TNF-*α* level in the kidney of the rats in the shock group was significantly increased than that in the sham group. Mesenteric lymph drainage reduced the TNF-*α* level, but this level in the drainage group was higher than that in the sham group (Figure 9).

## 4. Discussion

The factors influencing acute renal failure (ARF) are commonly categorized as prerenal, intrinsic and postrenal. In general, prerenal ARF is caused by kidney hypoperfusion resulting from hemorrhage, trauma, and hypotension. The early phase of prerenal ARF is a functional response; therefore, renal function returns to normal levels within one day to two days after etiopathogenesis and inducement are removed. However, renal function changes from a functional response to organ damage because of the ischemic necrosis of the renal tissues, along with the continuous hypoperfusion of the kidney. To improve the efficiency of early diagnosis and treatment, clinicians recommend the monitoring of the quality of acute dialysis, in which ARF is replaced with AKI, including mild acute renal insufficiency [17].

Based on a model of AKI induced by sustained hypoperfusion, the present study investigated the pathogenesis and intervention of AKI from the return of shock mesenteric lymph to the systemic circulation. The results showed that the sustained hypoperfusion of the kidney following hemorrhage caused structural damage and increased urea and Cre concentrations. This increase resulted from renal insufficiency; the hypermetabolic state induced by sustained hypotension also contributed to the increased urea and Cre concentrations. Lymph drainage from 1 h to 3 h after the duration of shock inhibited necrosis in the epithelial cells of the renal tubules and reduced urea concentration in the plasma. In the shock group, the Cre level in the plasma did not decrease after lymph was drained. These changes occurred because the Cre concentration remained in normal ranges, although this concentration increased after shock; furthermore, the removal effect of mesenteric lymph drainage on Cre concentration was not significant.

The extensive release of trypsin, a proteolytic enzyme, from damaged cells further digests the peripheral tissue protein and exacerbates cell injury. Therefore, trypsin levels can be used as a marker of cell damage [18]. In this study, trypsin activity in the plasma increased significantly in the rats of the shock group. The increased trypsin activity could occur as a consequence of cell injury and exacerbate cell injury. The mesenteric lymph was drained out of the systemic circulation after shock; as a result, trypsin activity in the plasma decreased. This result possibly occurred because cell damage was ameliorated by reducing injurious factors containing shock mesenteric lymph. This result could likely occur by reducing the return of trypsin carried in the mesenteric lymph. However, this hypothesis should be further confirmed. Trypsin is the main factor causing gut injury during hemorrhagic shock [19]. Therefore, the inhibition of trypsin activity may contribute to the preservation of intestinal barrier function. Such inhibition can also reduce the translocation of gut-derived injurious factors via the mesenteric lymphatic pathway.

ICAM-1 is mainly distributed in endothelial cells, epithelium, monocyte, lymphocytes, and dendritic cells [20]. Under stressful conditions, such as hemorrhage, trauma, and infection, the expression and synthesis of ICAM-1 is upregulated rapidly, thereby inducing neutrophils to bind to vascular endothelial cells and exacerbate inflammatory response and microcirculation dysfunction [21]. The present result suggested that the increased ICAM-1 in the kidney after 3 h of hypotension triggered neutrophil sequestration; as a result, inflammatory response and free radical injury are exacerbated. This finding is consistent with the increased contents of TNF-*α* and MDA in the kidney. TNF-*α* initially appears and functions as a mediator after the cells are challenged by stress factors [8, 9]. The excessive free radicals react with the unsaturated fatty acid located in the membrane of tissue cells to produce massive lipid peroxides, which destroy the structural integrity of cell membrane and organelles and induce a cascade of free radical injury. Moreover, the free radicals can result in peptide cross-link, DNA breakage, and structural change in cells [8, 9]. Therefore, the excessive increase in ICAM-1 following shock challenge is an important contributor of inflammatory response and cell injury in the kidney.

The study also found that the contents of ICAM-1 were decreased by the postshock mesenteric lymph drainage, which may inhibit the inflammatory response and cell injury of the kidney. This assumption is confirmed by the results of TNF-*α* and MDA changes in the kidney. These results also indicated that the mechanism of drainage in the shock mesenteric lymph attenuating kidney injury is related to the decrease in ICAM-1. This mechanism is also associated with inflammatory response suppression and free radical injury. We also found that the TNF-*α* level in the renal tissue of the rats in the drainage group increased compared with that in the sham group, although the postshock mesenteric lymph drainage decreased the TNF-*α* level of the shocked kidney. These results also suggested that other candidates could be involved in the increase in TNF-*α* in the shocked kidney. Thus, we further observed the change in RAGE in the kidney during shock.

The distribution of RAGE exhibits tissue heterogeneity and is mainly located in the monocyte-macrophage system of peripheral blood, vascular endothelial cells, alveolar epithelial cells, neurons, and tumor cells. RAGE is a 35 kD transmembrane receptor of the immunoglobulin superfamily and can bind advanced glycation end-products (AGEs). The combination of RAGE with AGEs initiates a series of cell injurious events and contributes to tissue damage in various inflammatory mediators inducing inflammatory response [22–24]. The present study showed that RAGE was increased in the kidney after shock but was decreased by the drainage of shock mesenteric lymph. Combined with the change in TNF-*α*, the results of this study suggested that the mechanism of shock mesenteric lymph drainage attenuating kidney injury is related to decreased RAGE. The results showing the levels of RAGE in the drainage group decreased compared with those in shock group; by comparison, these levels increased compared with those in the sham group. These results are consistent with the change in TNF-*α*. These findings further suggested that RAGE may be important in shock mesenteric lymph mediated kidney injury. However, this hypothesis should be further verified.

In addition to inflammatory response and free radical damage, the ischemic injury of the kidney induced by sustained hypoperfusion is important in the pathogenesis of shock. Therefore, we observed the changes in 2,3-DPG related to oxygen supply and LA related to energy metabolism in the kidney. 2,3-DPG levels are important factors influencing the affinity of hemoglobin and oxygen to erythrocytes. Such a decrease in 2,3-DPG levels enhances the affinity of hemoglobin and oxygen but reduces the oxygen supply to tissues. By contrast, the increase in 2,3-DPG attenuates the affinity of hemoglobin and oxygen; this increase is also conducive to the oxygen supply. The present study showed that the 2,3-DPG levels in shocked rats increased compared with those in the sham group. This result indicated that the oxygen supply of shocked erythrocytes to tissues increased. This change that occurred in erythrocytes may be one of the compensatory responses in the body. However, LA concentration in the shocked kidney was higher than that of the sham group. This result suggested that the disturbance of oxygen supply to the kidney was partially corrected, although the function of erythrocytes to provide oxygen supply increased. After shock mesenteric lymph drainage, 2,3-DPG concentrations further increased. As a result, oxygen supply was further improved in the circulating blood and it was beneficial to aerobic metabolism of cells. The decrease in LA concentrations in the drainage group indicated that aerobic metabolism in the kidney was restored when shock lymph was drained out of systemic circulation. Thus, the mechanism of shock mesenteric lymph drainage attenuating kidney injury is associated with the improvement of oxygen supply and aerobic metabolism.

In summary, kidney injury following sustained hypotension is associated with the return of shock mesenteric lymph to systemic circulation. The mechanism is also related to the decreased activity of trypsin and the suppression of inflammation response. The improvement of energy metabolism and attenuation of lactic acidosis are also responsible for this change. Therefore, the therapeutic strategy targeting shock mesenteric lymph may provide new insights into kidney injury during severe shock. However, the components of posthemorrhagic shock mesenteric lymph should be further examined.
